# Advances and Challenges in Prostate Cancer Diagnosis: A Comprehensive Review

**DOI:** 10.3390/cancers17132137

**Published:** 2025-06-25

**Authors:** Emil Kania, Maciej Janica, Miłosz Nesterowicz, Wojciech Modzelewski, Mateusz Cybulski, Jacek Janica

**Affiliations:** 1 Department of Urology, Śniadeckiego Voivodeship Hospital in Bialystok, 26 Sklodowskiej-Curie St., 15-278 Bialystok, Poland; 2 Department of Radiology, Śniadeckiego Voivodeship Hospital in Bialystok, 26 Sklodowskiej-Curie St., 15-278 Bialystok, Poland; 3 Department of Radiology, Medical University of Bialystok, 24A Sklodowskiej-Curie St., 15-276 Bialystok, Poland; 4 Department of Paediatric Radiology, Medical University of Bialystok, 17 Waszyngtona St., 15-274 Bialystok, Poland; 5 Department of Integrated Medical Care, Medical University of Bialystok, 7A Sklodowskiej-Curie St., 15-096 Bialystok, Poland

**Keywords:** prostate cancer, early detection, prostate-specific antigen (PSA), multiparametric magnetic resonance imaging (mpMRI), PI-RADS, transrectal ultrasonography (TRUS), prostate biopsy, targeted biopsy, fusion biopsy, combined fusion biopsy

## Abstract

Prostate cancer is the most commonly diagnosed malignancy in men and continues to be a leading cause of cancer-related mortality. Accurate and timely diagnosis is essential for distinguishing clinically significant tumors from indolent lesions and for informing treatment decisions. Multiparametric magnetic resonance imaging (mpMRI) has revolutionized prostate cancer detection by enabling precise lesion localization, risk stratification, and improved biopsy targeting. Fusion biopsy, which combines mpMRI findings with real-time transrectal ultrasonography (TRUS), has emerged as a highly effective method for sampling suspicious lesions. This review provides an integrated anatomical, epidemiological, technical, and clinical overview that highlights the evolving role of fusion biopsy in contemporary prostate cancer diagnostics. We also explore emerging strategies such as penumbra-targeted sampling, discuss ongoing clinical challenges, and examine the impact of biopsy underestimation and lack of standardization. Compared to conventional systematic biopsy, mpMRI-TRUS fusion biopsy improves the detection of clinically significant prostate cancer while reducing the overdiagnosis of low-risk tumors. To our knowledge, few recent reviews have comprehensively synthesized current clinical guidelines, emerging biopsy techniques, and future directions within a single narrative. mpMRI-TRUS-guided fusion biopsy represents a major advancement in the prostate cancer diagnostic pathway, promoting precision oncology by reducing overtreatment and facilitating individualized patient care. This review aims to assist clinicians in adopting biopsy innovations that enhance diagnostic accuracy and improve patient outcomes.

## Simple Summary

Prostate cancer is the most common cancer in men and can range from harmless to life-threatening. Accurately identifying which cases need treatment and which do not is essential for preventing unnecessary side effects. Traditional methods like ultrasound-guided biopsies often miss important tumors or detect harmless ones, leading to overtreatment. A newer method called fusion biopsy combines detailed MRI images with real-time ultrasound, allowing doctors to target the most suspicious areas more precisely. This approach improves cancer detection and reduces harm from unnecessary procedures. Our review explains how this technique works, how it compares to older methods, and what challenges still remain. We also discuss new developments that may further improve diagnosis in the future. Our goal is to help doctors make better-informed decisions and move toward more personalized care for men with suspected prostate cancer.

## 1. Introduction

Prostate cancer (PCa) is the second most commonly diagnosed cancer and the fifth leading cause of cancer-related death in men worldwide, with an estimated 1.41 million new cases and 375,000 deaths reported in 2020 [1]. In Poland, PCa ranks first among male cancers, with 17,638 new cases and 5618 deaths recorded in 2019 [2]. PCa incidence increases with age, contributing to its high prevalence in aging populations. In many cases, the disease is diagnosed at an advanced stage, which increases the risk of metastasis and disease-specific mortality. Both the disease and its treatment-related side effects significantly impair the quality of life and contribute to a substantial public health burden [1].

Initiating PCa treatment requires clinical suspicion, elevated tumor markers, suggestive imaging findings, and histopathological confirmation of malignancy via prostate biopsy. Although often asymptomatic in its early stages, PCa can present with nonspecific lower urinary tract symptoms (LUTS), hematuria, or hematospermia. These symptoms more commonly result from benign conditions—including benign prostatic hyperplasia (BPH), bladder outlet obstruction, urethral stricture, urinary tract infections, prostatitis, and chronic pelvic pain syndrome—which may hinder early detection due to their overlap with malignancy-related signs. In some cases, bone pain may constitute the initial manifestation of PCa, as bones and lymph nodes are common sites of metastatic spread. When detected early, PCa is often treatable with curative intent. This underscores the importance of improving early detection strategies, which represent a central focus of this review [3,4].

### 1.1. Structure and Function of the Prostate Gland

**Figure 1:**
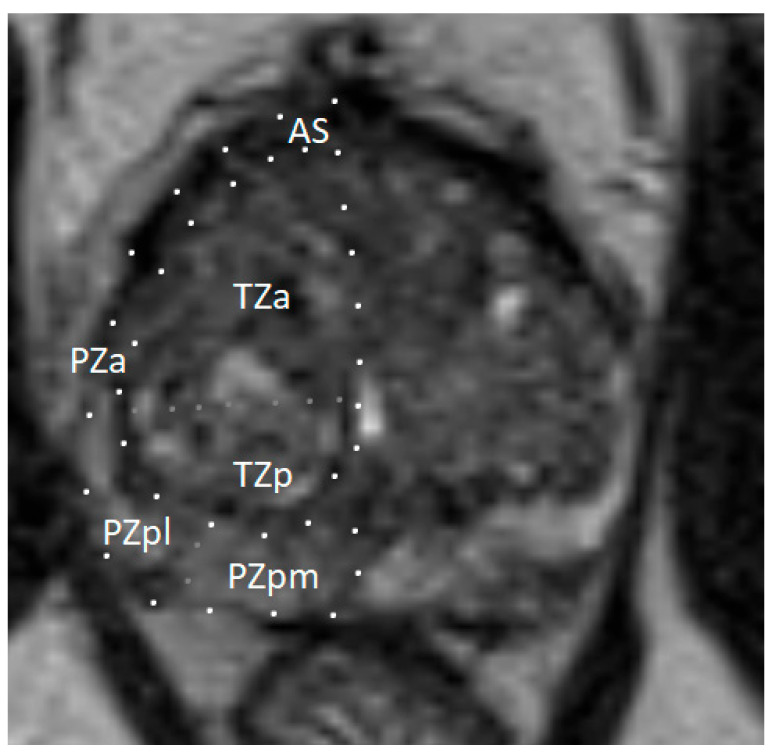
Zonal anatomy of the prostate gland on axial multiparametric magnetic resonance imaging (mpMRI). Adapted from the authors’ own materials, based on Yacoub and Oto [7]. a: anterior; AS: anterior [fibromuscular] stroma; p: posterior; pl: posterior lateral; pm: posterior medial; PZ: peripheral zone; TZ: transition zone.

The prostate gland is a small, walnut-sized exocrine organ. It is located in the lesser pelvis, between the bladder and the urogenital diaphragm, near the external urethral sphincter. The prostate consists of fibromuscular stroma and glandular elements, with excretory ducts lined by epithelial cells. The gland surrounds the proximal portion of the urethra, called the prostatic urethra, which measures approximately 4 cm in length. The vasa deferentia enter the prostate posteriorly, joining the urethra at the level of the ejaculatory ducts. Anatomically, the prostate consists of two lateral lobes and, in some individuals, a prominent median lobe [5]. The prostate is divided into peripheral, transition, and central zones—key anatomical regions for imaging-based lesion localization (see Figure 1). Approximately 80% of PCa cases originate in the peripheral zone, while 20–25% arise from the transition zone. Cancers in the central zone are uncommon and are usually extensions of tumors originating in the transition zone [6].

The prostate is surrounded by a fibromuscular capsule containing smooth muscle fibers, which contract during ejaculation to expel seminal fluid into the urethra. Prostatic secretions, which account for about 30% of ejaculate volume, enhance sperm motility and viability. These secretions contain prostate-specific antigen (PSA), which plays a key role in semen liquefaction and facilitates sperm motility. PSA is also believed to aid sperm penetration by degrading cervical mucus, although this role remains less clearly defined [8].

### 1.2. Epidemiology of Prostate Cancer

Globally, PCa is the second most frequently diagnosed malignancy and ranks as the fifth leading cause of cancer-related mortality among men. In 2020, an estimated 1.41 million new cases of PCa were diagnosed worldwide, resulting in approximately 375,300 deaths [1]. The median age at diagnosis of PCa in the United States of America (USA) is approximately 67 years [9]. Available data suggest that PCa incidence and mortality rates are declining or stabilizing in many high-income countries, reflecting advances in early detection and treatment. Nevertheless, PCa continues to pose a major public health challenge, particularly in the context of aging populations and disparities in healthcare access associated with socioeconomic development [10]. In Poland, 17,638 new cases and 5618 PCa-related deaths were reported in 2019, underscoring the disease’s national burden. PCa is the most frequently diagnosed cancer in Polish men, accounting for 21% of male malignancies and contributing to 10% of all cancer-related deaths [2].

In the USA, the five-year relative survival rate for men diagnosed with localized or regional PCa remains close to 100%, according to the most recent data from 2015 to 2021. However, this rate declines markedly to approximately 36% for cases diagnosed with distant metastases. Across all stages, the overall five-year relative survival rate for PCa in the USA is approximately 97% [11,12]. In Poland, data from 2015 to 2019 indicate a five-year relative survival rate of approximately 61.5% for PCa, which remains significantly lower than rates observed in Western Europe [13,14]. Earlier data (2010–2014) reported a survival rate of 78%, indicating a possible stagnation or decline in survival outcomes. Moreover, between 2015 and 2020, PCa mortality in Poland increased by 18%, compared to a 7% decrease across the European Union (EU) during the same period [15]. These disparities emphasize the urgent need to improve early detection, implement effective national screening programs, and expand access to treatment in the Polish healthcare system.

### 1.3. Etiology of Prostate Cancer

Although the etiology of PCa is not fully understood, both genetic predispositions and environmental factors are known to contribute to disease development. Men with first-degree relatives affected by prostate or breast cancer exhibit a markedly elevated risk of PCa, indicative of a hereditary component [16]. However, confirmed hereditary PCa (HPCa) accounts for only a minority of cases. HPCa typically presents 6 to 7 years earlier than sporadic cases, although its clinical course and aggressiveness are generally comparable [17].

Genetic alterations affect both the risk of developing PCa and the likelihood of more aggressive tumor phenotypes [18]. Approximately 15–17% of men with PCa carry germline mutations, most commonly involving pathogenic variants in BRCA2 (5%), CHEK2 (2%), ATM (2%), and BRCA1 (1%) [19]. Mutations in BRCA1 or BRCA2 are strongly associated with high-risk PCa, advanced stage at diagnosis, lymph node invasion (LNI), and distant metastases [20,21]. Among BRCA1 or BRCA2 mutation carriers, PSA-based screening often detects PCa at a younger age and a more advanced stage than in non-carriers [22].

Advancing age remains one of the strongest risk factors for PCa. Men over 50—or over 45 in those with a family history or African ancestry—are at increased risk [23,24,25]. A systematic review of autopsy studies found that the prevalence of subclinical PCa is about 5% in men under 30 years of age, increasing incrementally with age to approximately 59% in men over 79 [26].

These findings underscore the complex interplay between hereditary predisposition, age-related biological changes, and population-specific risk factors in the pathogenesis of PCa.

### 1.4. Pathomorphological Classification of Prostate Cancer

A definitive diagnosis of PCa cannot rely solely on clinical presentation, physical examination, imaging studies, or laboratory parameters such as serum PSA levels. Histopathological evaluation of prostate biopsy specimens remains the gold standard for confirming the diagnosis.

Adenocarcinoma accounts for over 95% of prostate malignancies, is frequently multifocal, and exhibits histological heterogeneity. Clinical staging is established using the tumor–node–metastasis (TNM) system [27,28].

The Gleason grading system remains the standard for the histopathological evaluation of tumor differentiation in PCa. It evaluates architectural patterns of glandular differentiation, including tubule formation, epithelial integrity, and the tumor’s interface with adjacent normal tissue [29].

**Figure 2:**
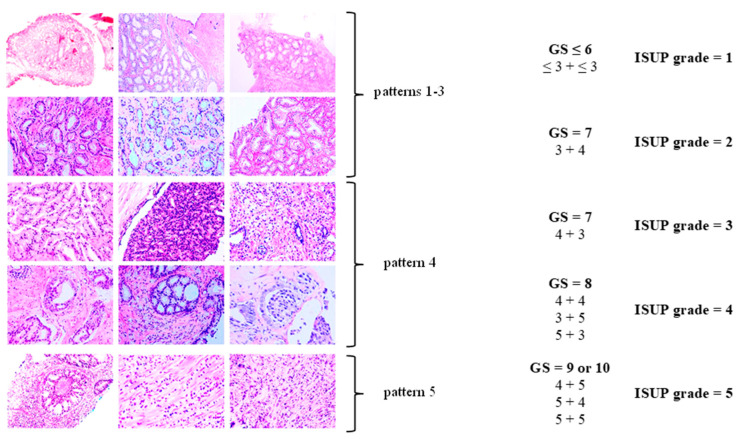
Histopathological classification of prostate cancer (PCa). Modified according to Linkon et al. [31] under Creative Commons Attribution (CC BY) 4.0 International License available at https://creativecommons.org/licenses/by/4.0/. GS: Gleason score; ISUP: International Society of Urological Pathology.

The Gleason system defines five histologic patterns, ranging from pattern 1 (well-differentiated) to pattern 5 (poorly differentiated). Given the heterogeneity of adenocarcinoma, the Gleason score (GS) reflects the sum of the most common and the second-most-common histologic patterns. The resulting GS, calculated as the sum of the primary and secondary patterns, is then translated into an International Society of Urological Pathology (ISUP) grade group [27,30] (see Table 1, Figure 2).

Based on PSA levels, GS, and tumor grade, patients are stratified into risk categories to estimate recurrence risk following radical therapy [30] (see Table 2). Following definitive therapy, a rise in PSA in the absence of clinical or radiographic evidence of disease is referred to as biochemical recurrence (BCR).

## 2. Initial Diagnosis of Prostate Cancer

The initial diagnostic assessment for PCa typically includes serum PSA measurement and digital rectal examination (DRE). Although repeated PSA testing and DRE are recommended, the optimal frequency of these assessments has not been established [32]. Current guidelines advocate a risk-adapted screening strategy based primarily on baseline PSA values. Men with PSA levels < 1 ng/mL at age 40 or <2 ng/mL at age 60 are considered at low long-term risk for metastatic PCa or disease-specific mortality [30,33,34]. The guidelines recommend that men at increased risk undergo PSA testing every two years, while those with low baseline PSA and no family history may extend the interval to as long as eight years [30,35].

There is no universally accepted age at which routine PCa screening should be discontinued. Clinical decisions regarding the cessation of screening should be individualized, taking into account comorbid conditions and life expectancy [30,36]. Men with a life expectancy under 10 years are generally unlikely to benefit from PCa treatment; however, those with high-grade disease (e.g., ISUP grade group 4 or 5) may still derive meaningful benefit from active intervention, even with limited life expectancy [30,37].

Contemporary diagnostic protocols increasingly incorporate multiparametric magnetic resonance imaging (mpMRI), which improves the detection and localization of malignant lesions [38]. Findings from PSA testing, DRE, and mpMRI inform the decision to proceed with prostate biopsy.

PCa can be classified as clinically significant or insignificant, although definitions of this distinction vary across the literature. PCa is considered clinically significant if any of the following criteria are met: a GS of 3 + 4 = 7 or higher (ISUP ≥ 2), a tumor focus measuring > 0.5 cm^3^, or evidence of extraprostatic extension (EPE). In contrast, PCa with a GS of 3 + 3 = 6 (ISUP = 1) is considered clinically insignificant [39,40,41,42].

A study of men with ISUP grade 1 PCa found that EPE was very rare (<0.5%), and no cases of seminal vesicle invasion (SVI) or LNI were observed [43,44]. Therefore, ISUP grade 1 PCa is considered clinically insignificant, as it typically exhibits indolent behavior despite its histological malignancy.

Distinguishing between clinically significant and clinically insignificant PCa is essential, as low-risk cancers—often not associated with mortality or reduced quality of life—are common [26]. Without appropriate risk stratification, men with low-risk cancer may undergo unnecessary radical treatment, such as surgery or radiotherapy, which may provide limited oncologic benefit in such cases. These interventions can result in significant adverse effects, including erectile dysfunction or urinary incontinence after radical prostatectomy. Overtreatment of clinically insignificant PCa has been criticized as a major drawback of widespread screening, which involves monitoring PSA concentrations in asymptomatic men [18].

In practice, distinguishing whether a case involves clinically significant or clinically insignificant PCa is challenging. The primary challenge is the underestimation of cancer aggressiveness, which may lead to misclassification of high-grade tumors as low-risk. Underestimation may result from diagnostic error, leading to inadequate assessment and an inappropriate therapeutic strategy.

### 2.1. Rectal Palpation

DRE permits the evaluation of the posterior aspect of the prostate, which is accessible via the rectum. Approximately 70% of PCa cases arise in the peripheral zone, which is generally accessible during DRE. However, when used in isolation, DRE demonstrates limited diagnostic performance, with sensitivity and specificity < 60%. DRE is inherently subjective, characterized by high interobserver variability and inconsistent interpretation among clinicians [45]. Consequently, DRE should not be employed as a standalone diagnostic tool but rather as part of a multimodal assessment strategy.

### 2.2. Prostate-Specific Antigen

PSA is a serine protease belonging to the kallikrein family, secreted predominantly by the epithelial cells of the prostate gland [46,47,48]. Under physiological conditions, PSA is present in high concentrations within prostatic tissue and seminal fluid, with only minimal amounts entering the systemic circulation [49,50]. Serum PSA concentrations typically rise in the presence of PCa, making PSA a valuable biomarker for initial diagnostic evaluation [47].

Although malignant prostate cells often produce less PSA per cell than benign epithelial cells, architectural disruption facilitates increased PSA leakage into the bloodstream. As cancer infiltrates the tissue, uncontrolled growth and abnormal cell differentiation increase PSA leakage from the cells into the extracellular fluid and bloodstream. This increased leakage is primarily attributed to the loss of the basal cell layer in malignant glands, which normally limits PSA passage into surrounding tissues and the bloodstream [51]. Elevated serum PSA remains the primary biochemical indicator prompting suspicion of prostate malignancy. Given that PSA levels physiologically rise with age, age-specific reference ranges may improve diagnostic accuracy [51,52].

Several benign and inflammatory conditions can also elevate serum PSA levels due to increased disruption of the prostatic epithelium. PSA levels may also rise temporarily after medical procedures such as DRE, cystoscopy, prostate biopsy, or transurethral resection of the prostate. Ejaculation and prostatic massage may also transiently elevate PSA levels. Although BPH can elevate PSA levels, a larger prostate volume is generally associated with a lower likelihood of clinically significant PCa [53]. PSA concentrations can be reduced by certain medications, particularly 5-alpha-reductase inhibitors used for BPH.

Serum PSA exists in two primary forms, free PSA (fPSA) and protein-bound PSA, the sum of which constitutes total PSA (tPSA). In pathological conditions, the ratio between free and bound PSA is often altered. Malignant conditions are typically associated with a lower proportion of fPSA relative to tPSA when compared to BPH. Assessment of the f/tPSA ratio improves diagnostic specificity in distinguishing PCa from benign conditions. An f/tPSA ratio < 16% is generally considered suggestive of malignancy, with lower percentages correlating with an increased PCa probability [54].

PSA density (PSA-D), calculated as serum PSA level divided by prostate volume, serves as an additional risk stratification tool. Elevated PSA-D values, particularly ≥ 0.15 ng/mL/cm^3^ in men with prostate volumes under 50 cm^3^, are associated with an increased likelihood of ISUP grade ≥ 2 disease [55]. Conversely, a PSA-D < 0.09 ng/mL/cm^3^ is generally associated with a low probability of clinically significant cancer [56].

PSA kinetics—including PSA doubling time (PSA-DT) and PSA velocity (PSA-V)—further assist in assessing cancer aggressiveness and monitoring disease progression [57]. Serial PSA measurements are integral in post-treatment surveillance, with rising levels potentially signifying BCR.

### 2.3. Screening and Early Detection

Population-based screening involves a systematic evaluation of asymptomatic individuals to detect cancer early, aiming to reduce mortality, improve quality of life, and increase the likelihood of cure.

PSA-based screening has been shown to lower PCa-specific mortality in certain populations [36]. However, widespread PSA screening is associated with the overdiagnosis of indolent cancers and may subject patients to complications arising from unnecessary invasive procedures [58,59]. Studies estimate that 20% to 50% of screen-detected PCa cases represent clinically insignificant tumors [59]. A substantial proportion of men with PCa remain asymptomatic and would remain undiagnosed in the absence of screening [58]. Detecting low-risk PCa in asymptomatic individuals may lead to treatment-related complications without providing clear clinical benefit. However, the optimal way to reduce overtreatment is to separate the diagnostic process from the automatic initiation of therapy.

Screening for PCa is currently one of the most controversial topics in the urologic literature [60,61]. The results of randomized clinical trials on PSA screening are conflicting [36,62,63,64].

The European Association of Urology (EAU) recommends that asymptomatic men aged 55–69 consider PSA screening on an individual basis [30]. Prior to testing, men should engage in shared decision-making with their physicians, considering personal values, preferences, and clinical context. In this decision-making process, both the patient and physician should consider family history, ethnicity, comorbidities, and life expectancy. PSA screening is not recommended without such discussion and is generally discouraged for asymptomatic men over 70 years of age [30,58,59].

### 2.4. Genomic Classifiers in Risk Stratification

In recent years, genomic classifiers have emerged alongside PSA, imaging, and histopathology to improve risk stratification in newly diagnosed PCa and to distinguish indolent from clinically significant disease. Three prominent assays—Decipher, Oncotype DX Genomic Prostate Score (GPS), and Prolaris—evaluate gene expression profiles in biopsy or prostatectomy tissue to predict tumor aggressiveness and long-term outcomes.

Decipher, a 22-gene classifier, has been validated extensively, demonstrating strong predictive ability for metastasis and PCa-specific mortality. For example, in a veteran cohort that included African American men, Decipher outperformed the Cancer of the Prostate Risk Assessment Postsurgical Score (CAPRA-S), achieving a concordance index (C-index) of 0.78 vs. 0.72 for metastasis and 0.85 vs. 0.81 for 10-year tumor-specific survival [65]. It also provides reliable risk predictions from biopsy specimens, with a C-index around 0.80–0.86 for 10-year metastasis risk, supporting its clinical utility in early diagnosis [66].

Oncotype DX GPS examines twelve genes involved in key pathways of prostate carcinogenesis and five reference genes, producing a score from 0 to 100. It predicts adverse pathology at prostatectomy, helps guide initial management decisions, and promotes a greater uptake of active surveillance (AS) in patients with low to intermediate risk [67,68].

Prolaris, which measures the expression of 31 cell cycle progression (CCP) genes normalized to housekeeping genes, provides a continuous CCP score correlated with 10-year progression and mortality risk. In real-world clinical practice, Prolaris results have altered management recommendations in approximately 50–65% of cases, particularly shifting borderline-risk patients toward AS [69].

A comprehensive review of PCa genomic tests from 2012 to 2021 concluded that all three tools—Decipher, Oncotype DX GPS, and Prolaris—consistently improve risk discrimination beyond traditional clinical factors [70]. Nonetheless, prospective data on long-term outcomes remain limited, and unresolved issues include cost-effectiveness, equitable access, and broader adoption in clinical guidelines. Decipher is currently the most robustly validated classifier, with inclusion in National Comprehensive Cancer Network (NCCN) guidelines (v1.2025) as a medically appropriate adjunct for both biopsy-only and post-prostatectomy decision-making [71].

Incorporating genomic classifier results into clinical workflows offers a more personalized assessment of a patient’s cancer biology. In men with low- or favorable intermediate-risk disease and a life expectancy of ten years or more, these tests may aid in selecting AS over immediate intervention. Conversely, in higher-risk or post-surgical contexts, genomic scores can guide adjuvant therapy decisions or selection for clinical trials.

### 2.5. Imaging Studies

Imaging studies play a key role in the diagnosis and management of PCa, allowing assessment of the prostate’s size, shape, zonal anatomy, and spatial relationship to surrounding structures. They assist in detecting pathological changes, estimating tumor burden, and evaluating EPE, SVI, and LNI—key elements in staging and treatment planning [72]. Imaging is also essential for guiding biopsy procedures and for identifying candidates for focal therapies or nerve-sparing surgery [73]. Various imaging modalities are used in PCa, each with its diagnostic value depending on the clinical context and disease stage.

#### 2.5.1. Transrectal Ultrasonography

Transrectal ultrasonography (TRUS) is a widely available, safe, and minimally invasive modality used for structural imaging of the prostate and real-time guidance of both transrectal and transperineal biopsy needles [74,75,76,77,78]. It provides useful information on prostate size, zonal anatomy, and echogenic alterations suggestive of pathology—hypoechoic, isoechoic, or hyperechoic lesions—but gray-scale TRUS alone has limited diagnostic accuracy. Studies report a sensitivity ranging from 50 to 76% and a specificity of 76–94%, often dependent on operator skill and equipment quality [79]. Although approximately 60–80% of cancers appear hypoechoic, only 17–57% of hypoechoic lesions are malignant, resulting in a positive predictive value (PPV) of roughly 25–30% [75,79,80,81,82]. TRUS demonstrates limited accuracy in staging EPE or SVI, with a sensitivity ranging from 22 to 60% and a specificity of up to 88% [81,83,84].

#### 2.5.2. Computed Tomography

Computed tomography (CT) plays a restricted yet clinically valuable role in PCa staging. While its sensitivity for detecting clinically significant primary tumors remains low, CT is valuable for identifying EPE, LNI, and distant metastases. For lymph node assessment, CT shows a pooled sensitivity of approximately 42% and a specificity of around 82%. Some studies report a specificity as high as 97%, indicating its reliability in confirming nodal positivity; however, it carries a risk of false negatives for micrometastases. In detecting bone metastases, recent whole-body low-dose CT has shown 81–89.4% sensitivity and 96.6–98.5% specificity, making it useful for staging despite the potential to miss small lesions [85,86]. A broader literature review indicates that bone CT has a sensitivity of approximately 74% and a specificity of around 56% for detecting osteoblastic metastases, performing better than standard radiography, but it is still limited in identifying early intramedullary disease [87].

#### 2.5.3. Multiparametric Magnetic Resonance Imaging

In recent years, mpMRI has become an increasingly common tool in the diagnosis of PCa. It allows for precise anatomical imaging of the prostate gland and surrounding tissues, an accurate assessment of the size, location, and characteristics of tumor lesions, and for the detection of the presence of enlarged lymph nodes in the pelvis [88]. mpMRI helps detect EPE, SVI, and LNI, supporting surgical planning, extent determination, as well as neurovascular bundle preservation [88].

mpMRI includes anatomical T1- and T2-weighted images, diffusion-weighted imaging (DWI) with apparent diffusion coefficient (ADC) maps, and dynamic contrast enhancement (DCE) following intravenous contrast administration [89].

mpMRI is currently the most effective modality for local staging of PCa, offering significantly higher sensitivity and specificity than other imaging techniques [90].

Features seen on mpMRI correlate with the likelihood of malignancy in visualized lesions. To standardize interpretation, the PI-RADS classification was developed. It assigns a score from 1 to 5 to mpMRI-detected lesions, reflecting their likelihood of malignancy [91] (see Table 3). The system is designed to ensure consistent lesion characterization, enhance targeted biopsy precision, and inform treatment decisions. In PI-RADS, each mpMRI sequence is evaluated separately as part of the overall assessment. The overall PI-RADS score is based on the dominant sequence in the affected prostate zone. The final PI-RADS assessment corresponds to the dominant lesion (index lesion) with the highest score within the evaluated prostate zone.

Compared to other imaging modalities, mpMRI demonstrates superior performance in detecting clinically significant PCa. While TRUS offers basic anatomical guidance and remains a standard for biopsy targeting, it lacks the tissue characterization capabilities of mpMRI and has substantially lower sensitivity for clinically significant PCa, often resulting in missed lesions or unnecessary sampling. In contrast, mpMRI, particularly when combined with targeted biopsy techniques, increases the detection rate of clinically significant PCa and decreases the overdiagnosis of indolent tumors. For example, the Prostate Magnetic Resonance Imaging Study (PROMIS) reported that mpMRI achieved a sensitivity of 93%, whereas standard TRUS-guided biopsy (TRUS-Bx) reached only 48%, with mpMRI demonstrating a significantly lower false-negative rate. Similarly, CT is inferior to mpMRI in visualizing intraprostatic lesions due to its limited soft tissue contrast and is mainly reserved for detecting EPE or distant metastases. Overall, mpMRI provides the most comprehensive assessment of prostate anatomy and pathology, making it the preferred imaging modality for initial evaluation and biopsy planning in patients with suspected PCa [40,73,92,93].

PI-RADS classification should rely exclusively on mpMRI findings, without reference to PSA levels, DRE results, clinical history, or previous treatments. The detection rates of PCa with ISUP grade ≥ 2 based on PI-RADS scores are as follows: 9% (5–13%) for PI-RADS 1–2, 16% (7–27%) for PI-RADS 3, 59% (39–78%) for PI-RADS 4, and 85% (73–94%) for PI-RADS 5 [94].

**Figure 3:**
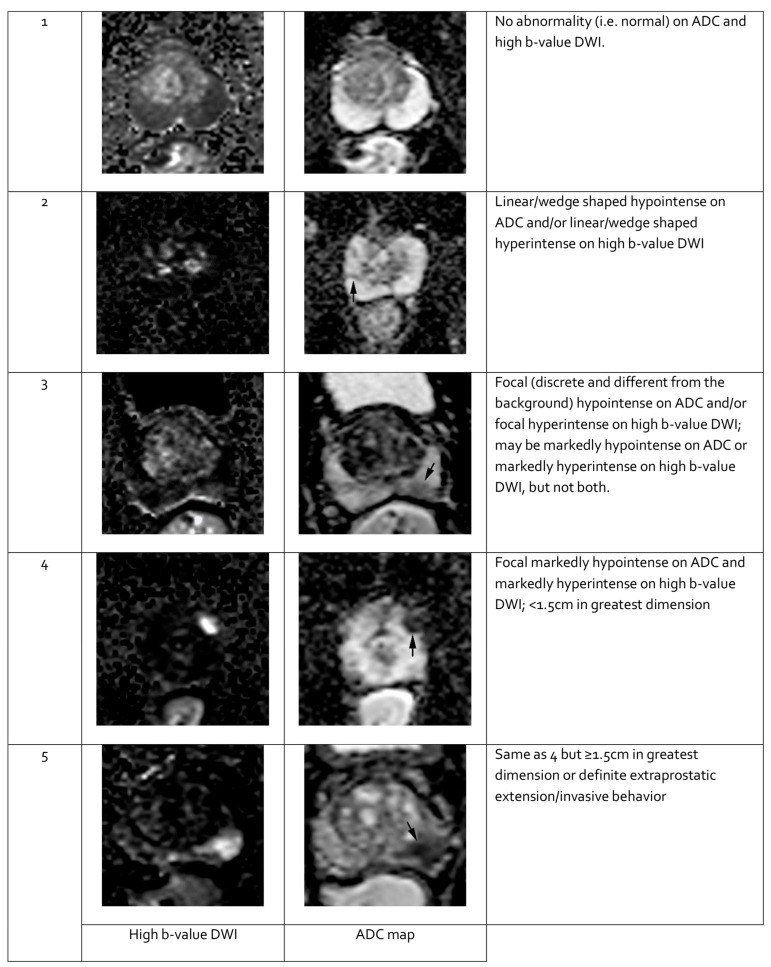
Representative images of peripheral zone lesions of the prostate gland with various Prostate Imaging—Reporting and Data System (PI-RADS) scores. Reprinted from PI-RADS v2.1 [91] under a Creative Commons Attribution-NoDerivatives (CC BY-ND) 4.0 International License available at https://creativecommons.org/licenses/by-nd/4.0/. ADC: apparent diffusion coefficient; DWI: diffusion-weighted imaging. Black arrows indicate lesions in the respective PI-RADS scores.

**Figure 4:**
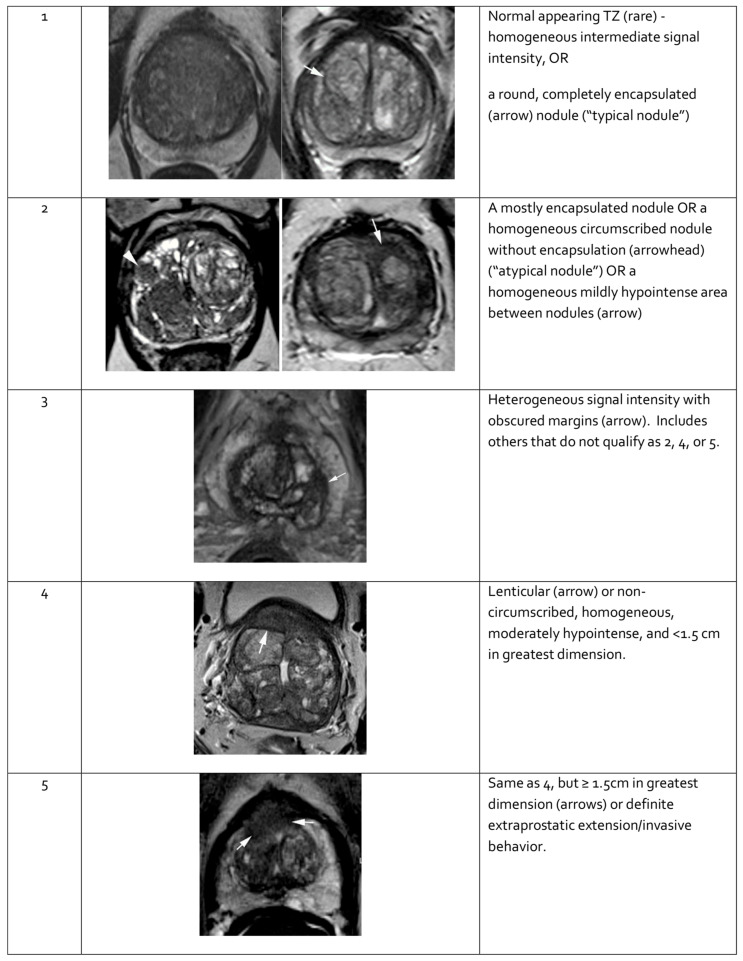
Representative images of transition zone lesions of the prostate gland with various Prostate Imaging—Reporting and Data System (PI-RADS) scores. Reprinted from PI-RADS v2.1 [91] under a Creative Commons Attribution-NoDerivatives (CC BY-ND) 4.0 International License available at https://creativecommons.org/licenses/by-nd/4.0/. TZ: transition zone.

When evaluating a lesion detected by mpMRI, its anatomical location is critical. T2-weighted images are most informative for lesions in the transition zone, while DWI is dominant for assessing the peripheral zone. Lesion measurements should be performed using the most informative imaging sequence for the specific prostate zone. Representative mpMRI images corresponding to PI-RADS scores are shown in Figure 3 and Figure 4. mpMRI is more effective in detecting clinically significant PCa lesions in the peripheral zone than in the transition zone. Given the growing number of mpMRI examinations, there is a trend toward simplifying protocols by omitting DCE. DCE plays a relatively minor role in primary PCa diagnosis and is associated with potential side effects, longer procedure time, and higher cost [95]. The role of DCE is more significant in the evaluation of local recurrences of cancer after radical treatment [96].

**Figure 5:**
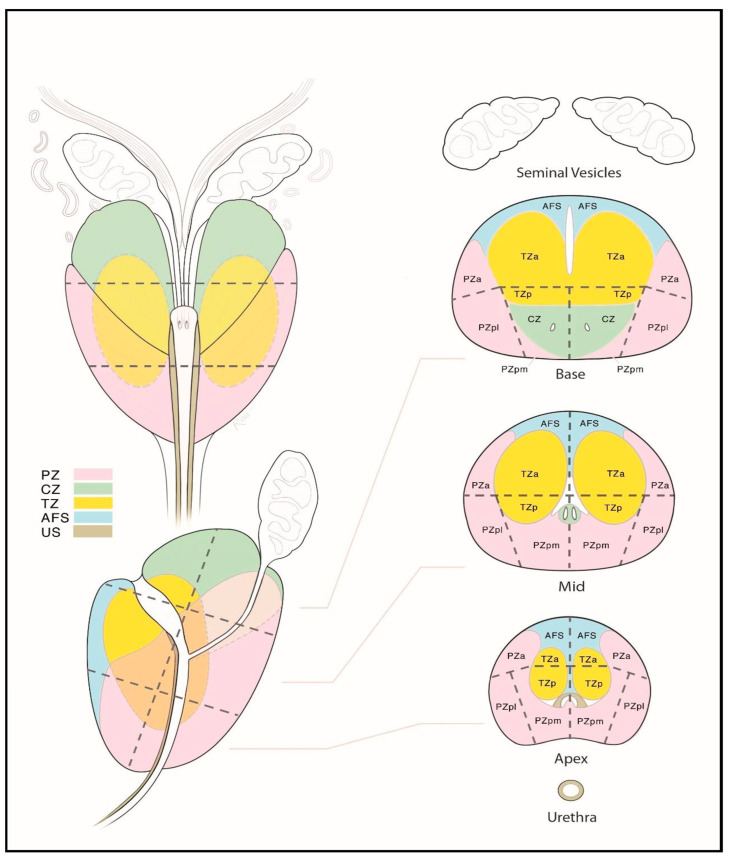
Sector map of the prostate gland illustrating a standardized localization of suspicious lesions on multiparametric magnetic resonance imaging (mpMRI). Reprinted from PI-RADS v2.1 [91] under a Creative Commons Attribution-NoDerivatives (CC BY-ND) 4.0 International available at https://creativecommons.org/licenses/by-nd/4.0/. a: anterior; AFS: anterior fibromuscular stroma; CZ: central zone; p: posterior; pl: posterior lateral; pm: posterior medial; PZ: peripheral zone; US: [external] urethral sphincter; TZ: transition zone.

To facilitate a consistent localization of pathological lesions within the prostate gland, it is recommended to use a sectoral map (see Figure 5). Dividing the prostate gland and adjacent structures into sectors standardizes reporting and enables radiologists, urologists, pathologists, and other specialists to precisely localize findings on mpMRI [91,97,98]. Knowing the tumor’s position relative to the prostate surface and its distance from adjacent structures—such as neurovascular bundles, the external urethral sphincter, and the bladder neck—is essential for planning nerve-sparing radical prostatectomy. A sector map is a helpful tool in surgical planning [99,100]. Additionally, the sector map can serve as a valuable visual aid when discussing biopsy strategies and available treatment options for PCa with patients [101,102].

A comparison of mpMRI findings with histopathological examination of specimens obtained during radical prostatectomy demonstrates that mpMRI has good sensitivity for detecting and localizing PCa with ISUP grade ≥ 2, particularly for lesions > 10 mm in diameter [103]. mpMRI has a lower detection rate for ISUP grade 1 lesions compared to those of grade 2 or higher. It identifies < 30% of ISUP 1 cancers with a volume of <0.5 mL, thereby reducing the risk of detecting low-risk PCa and preventing overdiagnosis and overtreatment [104].

Despite PI-RADS implementation, inter-reader concordance and lesion reproducibility remain only moderate, limiting reliable use among less experienced radiologists [105,106].

Routine mpMRI in biopsy-naïve men may reduce the number of patients requiring biopsy—an invasive procedure—and decrease the detection of low-risk PCa. In men with prior negative biopsy results under ongoing surveillance, mpMRI enhances the detection and localization of intermediate- and high-risk PCa foci [107].

According to EAU guidelines, mpMRI examination of the prostate gland is recommended prior to the initial biopsy. If mpMRI shows the presence of lesions with a PI-RADS score of 3 or higher, a prostate biopsy is recommended [30]. This approach maximizes the detection of clinically significant PCa in patients with visible lesions on mpMRI while minimizing biopsy-related risks in those without suspicious findings (PI-RADS 1 or 2) [107,108].

In the absence of visible lesions on mpMRI, a risk-adapted strategy is recommended. Men without suspicious lesions on mpMRI and with PSA-D between 0.1 and 0.2 ng/mL may defer immediate biopsy in favor of follow-up and future risk reassessment. However, men with a PSA-D value > 0.2 ng/mL should undergo systematic biopsy despite the absence of lesions with a PI-RADS score ≥ 3 on mpMRI [30].

#### 2.5.4. Prostate-Specific Membrane Antigen–Positron Emission Tomography–Computed Tomography

In recent years, prostate-specific membrane antigen–positron emission tomography–CT (PSMA-PET-CT) has emerged as a valuable tool for the diagnosis and staging of PCa. Due to its high sensitivity and specificity, PSMA-targeted imaging enables the detection of metastases and local recurrence even at low PSA levels [109]. Several studies have evaluated the integration of PSMA-PET-CT into multiparametric risk stratification models and nomograms to improve diagnostic accuracy and predict clinically significant PCa [110,111]. These approaches may enhance biopsy selection and guide personalized treatment planning. Although still undergoing clinical validation, PSMA-PET-CT-based predictive models show promise as adjuncts to mpMRI and genomic testing in the diagnostic pathway of PCa [112]. Further prospective trials are warranted to define their role in routine clinical practice and optimize integration into diagnostic algorithms [113].

## 3. Prostate Biopsy

Prostate biopsy with histopathological evaluation of tissue specimens remains essential for diagnosing malignant PCa. The ideal diagnostic test for PCa should be minimally invasive and carry a low risk of adverse effects. It should maximize the identification of men who would benefit from treatment while minimizing the detection of low-risk PCa.

In current PCa diagnostic pathways, mpMRI is recommended prior to the initial biopsy, alongside DRE and serum PSA testing [30]. Incorporating mpMRI into the biopsy protocol and performing targeted sampling of mpMRI-visualized areas increases the detection of intermediate- and high-risk cancers while reducing low-risk cancer diagnoses. It also helps avoid unnecessary biopsies when no lesions are visible [39,114]. Men with no visible lesions on mpMRI are at low risk of harboring PCa with ISUP grade ≥ 2 [73].

**Figure 6:**
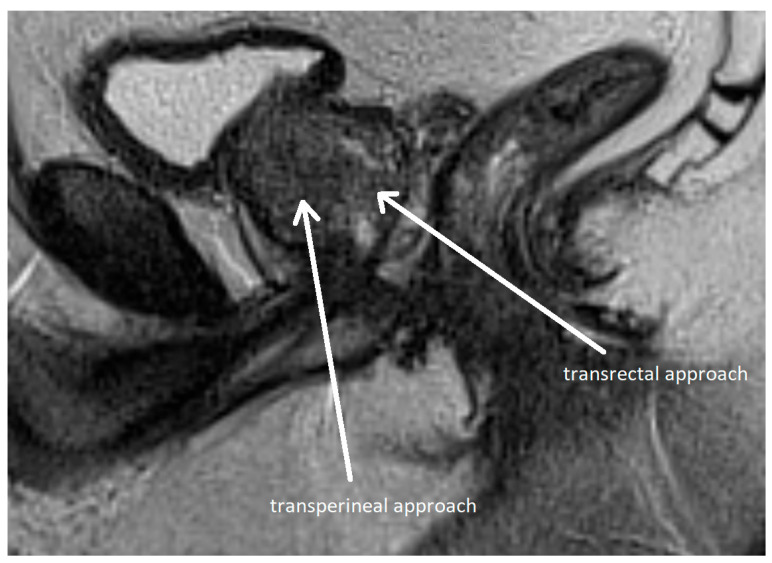
Transperineal and transrectal access routes for prostate biopsy visualized on sagittal multiparametric magnetic resonance imaging (mpMRI). The image illustrates needle trajectories through the perineum (transperineal approach) and rectum (transrectal approach) directed toward the prostate gland. The transrectal route is commonly used due to its simplicity and familiarity but carries a higher risk of infection. In contrast, the transperineal approach provides improved access to anterior and apical zones of the prostate and has a lower risk of sepsis. Adapted from the authors’ own materials, based on Chang et al. [118].

Methods of prostate biopsy can be divided by anatomic access into transrectal and transperineal (see Figure 6). Transperineal access is associated with a lower risk of infectious and hemorrhagic complications and better detection of anterior neoplastic lesions, although the procedure is more painful [115,116,117]. Therefore, the EAU recommends transperineal access as the preferred approach [30]. Biopsy techniques are classified as systematic or targeted, with the latter directed at mpMRI-identified lesions.

### 3.1. Historical Background

The first open perineal prostate biopsy was performed and described in 1926. Several years later, a fine-needle aspiration biopsy of the prostate was introduced. Initially, fine-needle aspiration biopsies were performed under digital rectal guidance. The 1980s marked a breakthrough in PCa diagnosis with the development of TRUS and the introduction of the biopsy gun, a device still in use today. Tissue samples are obtained by activating the gun’s spring-loaded mechanism, which rapidly advances the inner needle and then slides the cutting sheath over it. The advancement of imaging techniques and widespread availability of TRUS enabled more precise sampling from distinct prostate zones, establishing the systematic (sextant) biopsy as the new standard [119].

### 3.2. Systematic Biopsy

The most commonly used method of prostate biopsy today is systematic TRUS-Bx. It involves obtaining 8–12 core samples from regions of the prostate typically associated with cancer development. When biopsy sampling lacks precision—particularly in cases of persistent clinical suspicion of PCa—a repeat invasive procedure is often required [120].

### 3.3. Fusion Biopsy

With advances in prostate imaging and broader access to mpMRI, fusion biopsy has gained prominence in recent years. The goal of mpMRI prior to biopsy is to identify, localize, and characterize foci of clinically significant PCa. A diagnostic pathway that incorporates pre-biopsy mpMRI and targets identified lesions is more effective than systematic biopsy alone [40,95,121].

During targeted biopsy, tissue samples are obtained from mpMRI-detected suspicious lesions and are graded by a radiologist using the PI-RADS classification.

These include three types of targeted biopsy [122,123,124,125,126,127,128,129,130,131]:

1. Cognitive biopsy—after reviewing mpMRI images and the radiologic report, the operator mentally maps the lesion’s location and attempts to target the area under ultrasound guidance;
2. Direct mpMRI-guided biopsy (in-bore)—tissue sampling is performed within the MRI scanner, with real-time visualization and targeting of the lesion;
3. Fusion biopsy—dedicated software merges pre-acquired mpMRI images with real-time TRUS, allowing for a direct visualization of the marked lesions. The urologist then guides the biopsy needle to obtain tissue samples from these targets.

Fusion biopsy enables accurate co-registration of mpMRI-detected lesions with real-time ultrasound images. This allows for highly precise, targeted sampling and offers advantages over cognitive biopsy [126,127,128,129,130,131,132,133]. Fusion biopsy improves the detection of ISUP grade ≥ 2 PCa and may reduce the number of unnecessary procedures [39,114]. Fusion biopsy with mpMRI and ultrasound offers greater reproducibility, is less operator-dependent than cognitive biopsy, and provides real-time feedback on needle positioning during sampling [134]. In-bore mpMRI-guided biopsy has the highest diagnostic yield—up to 80%—but its time and cost requirements make it impractical for routine clinical use [135].

### 3.4. Limitations of Prostate Biopsy

**Figure 7:**
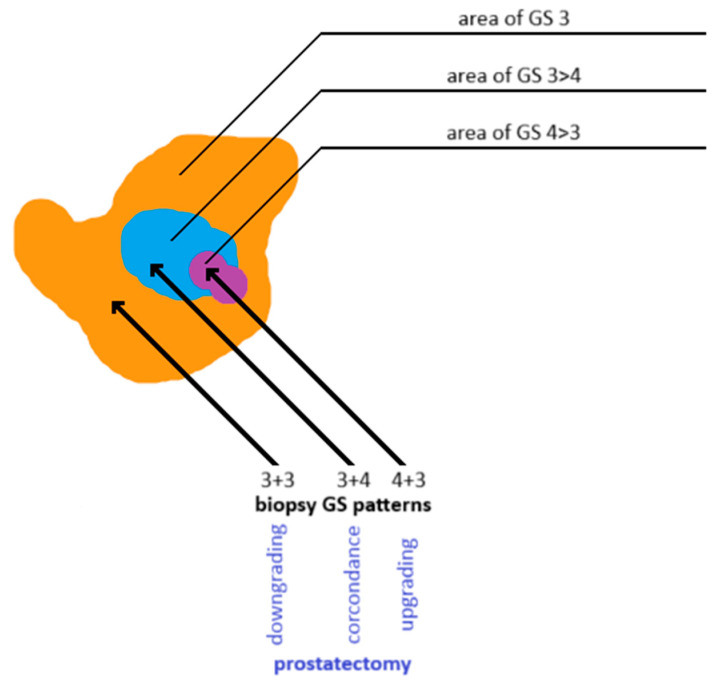
Discordance in Gleason grading between prostate biopsy cores and whole-gland specimens obtained after radical prostatectomy. Own work based on Goel et al. [140]. GS: Gleason score.

Underestimation of PCa grade on biopsy is a significant clinical concern, as it may result in the selection of an inappropriate therapeutic strategy. Although histopathologic evaluation of prostate tissue is crucial for assessing malignancy, the sampling technique can influence diagnostic accuracy. Tumor cellularity within a biopsy core may not accurately reflect the tumor’s overall histological heterogeneity. A biopsy core showing low-grade disease may be adjacent to—or partially contain—a higher-grade lesion (see Figure 7). Another challenge in defining clinically insignificant PCa is that ISUP grade 1 tumors may progress to higher grades over time, ultimately becoming clinically significant [103]. Underestimation may also result from false-negative biopsies due to an inadequate number of tissue cores. This occurs in approximately 30–80% of men with clinically significant PCa [126,127,128,129,130,131,136,137]. Obtaining 3–5 targeted cores per mpMRI-identified lesion improves sampling reliability and reduces inter-operator variability in detecting PCa [126,127,128,129,130,131,138,139].

If prostatectomy specimens reveal a higher Gleason grade than biopsy samples, the case is considered an upgrade, indicating a more aggressive disease identified postoperatively than initially detected on biopsy. Conversely, a lower Gleason grade on prostatectomy than on biopsy is termed downgrading [140,141].

Biopsy underestimation of PCa grade is associated with increased rates of positive surgical margins and BCR after radical prostatectomy [141]. Therefore, patients on AS for biopsy-confirmed ISUP grade 1 PCa may still be at increased risk for disease progression [142,143]. In contrast, downgrading may result in overtreatment, exposing the patient to unnecessary side effects and complications [142].

According to EAU guidelines, combined fusion biopsy (ComBx) includes both targeted and systematic mapping biopsies, with the latter obtained from regions not evaluated on mpMRI [30,41].

**Figure 8:**
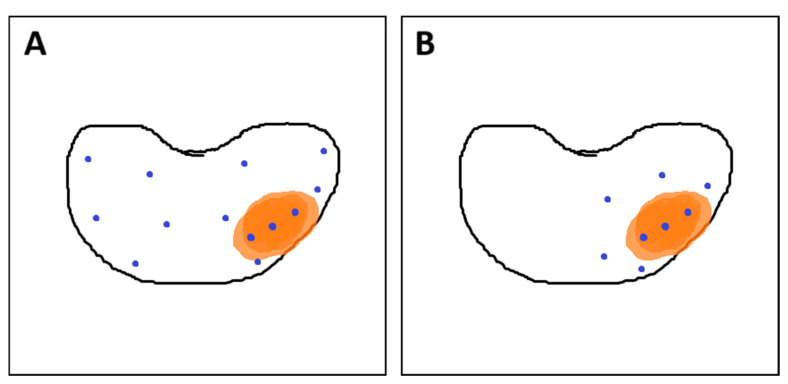
(**A**) Combined fusion biopsy (ComBx): targeted cores obtained from mpMRI-visible lesions, along with systematic mapping biopsies; (**B**) Targeted biopsy with additional samples from the 10 mm penumbra zone. Own work based on Brisbane et al. [145]. In both sections of the figure, the orange area represents the lesion, while the blue dots indicate the locations where biopsy cores were taken.

To minimize the number of biopsy cores required for accurate PCa grading, a strategy has been proposed in which a limited number of additional samples are obtained from a 10 mm penumbra surrounding the mpMRI-visible lesion. These penumbra cores are obtained in addition to targeted samples while omitting systematic mapping biopsies (see Figure 8). This approach may offer comparable accuracy in assessing PCa grade while reducing the risk of underestimation and minimizing patient discomfort associated with the invasive procedure [144].

## 4. Treatment of Prostate Cancer

In cases of low- to intermediate-risk PCa, AS may be an appropriate management strategy. This approach involves PSA monitoring, repeat imaging, and scheduled biopsies, allowing for the deferral of radical treatment without compromising oncologic outcomes [146]. If malignancy is detected on prostate biopsy, the patient is typically referred for definitive treatment, such as radical prostatectomy or radiation therapy [147]. Alternatively, patients with low-risk disease may be managed through AS [30,107,148]. AS is particularly suitable for low-risk PCa, which is typically indolent and associated with a low risk of progression or metastasis over many years. The strategy entails regular monitoring through serial PSA testing, imaging studies, and repeat biopsies to detect signs of disease progression [30]. Typically, PSA testing is performed every 3–6 months, DRE is conducted at least once a year, and repeat mpMRI is recommended every 12–18 months or earlier if there are signs of progression. Repeat biopsies are generally scheduled within the first 1–2 years and subsequently at intervals of 2–5 years, depending on individual risk stratification and disease progression [30,143,149]. This strategy enables safe deferral of definitive treatment while minimizing treatment-related morbidity, including urinary incontinence and erectile dysfunction.

In contrast, patients with intermediate- or high-risk PCa—typically defined by elevated PSA levels, GS ≥ 7, or more extensive disease—are generally recommended to undergo definitive treatment [150]. For patients with organ-confined disease, a life expectancy of more than 10 years is typically required to justify definitive treatment—except in cases of high- or very-high-risk disease, where treatment may still be warranted despite limited life expectancy [30,151,152]. Radical treatment offers the greatest benefit to patients with intermediate- or high-risk PCa and a favorable life expectancy. Treatment modalities may include radical prostatectomy, external beam radiation therapy (EBRT), or brachytherapy, sometimes combined with androgen deprivation therapy (ADT). In advanced or metastatic disease, systemic therapies such as novel androgen receptor inhibitors (e.g., enzalutamide, apalutamide) or chemotherapy (e.g., docetaxel) are commonly used [153]. Emerging technologies—including molecular profiling and AI-assisted imaging—are driving the development of more personalized treatment strategies.

## 5. Conclusions

Fusion biopsy guided by mpMRI and TRUS represents a significant advancement in the diagnostic pathway for PCa. This targeted approach achieves higher detection rates of clinically significant PCa compared to traditional systematic biopsy while reducing the overdiagnosis of indolent tumors. By improving diagnostic precision and reducing patient morbidity, mpMRI-TRUS fusion biopsy facilitates more personalized and effective treatment planning. To further improve outcomes, future efforts should focus on standardizing fusion biopsy protocols, enhancing operator training, and integrating advanced imaging biomarkers to improve risk stratification. With continued advancements in imaging technology, fusion biopsy is expected to become an essential component of precision oncology, optimizing the balance between early detection and the avoidance of overtreatment.

## Figures and Tables

**Table 1:** Histopathological classification of prostate cancer (PCa) according to the International Society of Urological Pathology (ISUP) Grade Group system. Modified from the European Association of Urology (EAU) [30] based on Braunhut et al. [27].

International Society of Urological Pathology (ISUP) Grade Group	Gleason Score (GS; the Sum of the Scores of the Most Dominant Pattern, as Well as the Second Most Common Pattern)
1	GS ≤ 6 (≤ 3 + ≤ 3)
2	GS = 7 (3 + 4)
3	GS = 7 (4 + 3)
4	GS = 8 (4 + 4, 3 + 5, or 5 + 3)
5	GS = 9 or 10 (4 + 5, 5 + 4, or 5 + 5)

GS: Gleason score; ISUP: International Society of Urological Pathology.

**Table 2:** Risk groups of localized and locally advanced prostate cancer (PCa) according to the European Association of Urology (EAU) [30]. Modified from the European Association of Urology (EAU) [30].

Low-Risk Group	Intermediate-Risk Group	High-Risk Group	
PSA < 10 ng/mL and GS < 7 (ISUP grade = 1), and T1–T2a	PSA = 10–20 ng/mL or GS = 7 (ISUP grade = 2 or 3), or T2b	PSA > 20 ng/mL or GS > 7 (ISUP grade = 4 or 5), or T2c	T3–T4 or N1
localized disease			locally advanced disease

GS: Gleason score; ISUP: International Society of Urological Pathology; N: regional lymph node category; PSA: prostate-specific antigen; T: primary tumor category.

**Table 3:** Risk of clinically significant prostate cancer (PCa) according to the Prostate Imaging—Reporting and Data System (PI-RADS) assessment categories based on multiparametric magnetic resonance imaging (mpMRI) findings [91]. Own work based on PI-RADS v2.1 [91].

PI-RADS Grade	Risk of Malignant Neoplasm
1	very low (clinically significant cancer is highly unlikely to be present)
2	low (clinically significant cancer is unlikely to be present)
3	intermediate (the presence of clinically significant cancer is equivocal)
4	high (clinically significant cancer is likely to be present)
5	very high (clinically significant cancer is highly likely to be present)

PI-RADS: Prostate Imaging—Reporting and Data System.

## Abbreviations

The following abbreviations are used in this manuscript:

ADC	apparent diffusion coefficient
ADT	androgen deprivation therapy
AS	active surveillance
BCR	biochemical recurrence
BPH	benign prostatic hyperplasia
C-index	concordance index
CAPRA-S	Cancer of the Prostate Risk Assessment Postsurgical Score
CCP	cell cycle progression
ComBx	combined fusion biopsy
CT	computed tomography
DCE	dynamic contrast enhancement
DRE	digital rectal examination
DWI	diffusion-weighted imaging
EAU	European Association of Urology
EBRT	external beam radiation therapy
EPE	extraprostatic extension
EU	European Union
fPSA	free prostate-specific antigen
f/tPSA	free-to-total prostate-specific antigen ratio
GPS	Genomic Prostate Score
GS	Gleason score
HPCa	hereditary prostate cancer
ISUP	International Society of Urological Pathology
LNI	lymph node invasion
LUTS	lower urinary tract symptoms
mpMRI	multiparametric magnetic resonance imaging
NCCN	National Comprehensive Cancer Network
PCa	prostate cancer
PI-RADS	Prostate Imaging—Reporting and Data System
PPV	positive predictive value
PROMIS	Prostate Magnetic Resonance Imaging Study
PSA	prostate-specific antigen
PSA-D	prostate-specific antigen density
PSA-DT	prostate-specific antigen doubling time
PSA-V	prostate-specific antigen velocity
PSMA-PET-CT	prostate-specific membrane antigen–positron emission tomography–computed tomography (PSMA-PET-CT)
SVI	seminal vesicle invasion
TNM	tumor–node–metastasis
tPSA	total prostate-specific antigen
TRUS	transrectal ultrasonography
TRUS-Bx	transrectal ultrasonography-guided biopsy
USA	United States of America
